# Whole-Genome Sequence of a Classical Swine Fever Virus Isolated from the Uttarakhand State of India

**DOI:** 10.1128/genomeA.00371-14

**Published:** 2014-05-08

**Authors:** Ravi Kumar, Kaushal Kishor Rajak, Tribhuwan Chandra, Ashish Thapliyal, Dhanavelu Muthuchelvan, Shashi Bhushan Sudhakar, Kuldeep Sharma, Arpit Saxena, Sachin D. Raut, Vinod Kumar Singh, Zubair Ahmad, Ajay Kumar, Dheeraj Chaudhary, Raj Kumar Singh, Awadh Bihari Pandey

**Affiliations:** a Division of Virology, Indian Veterinary Research Institute (IVRI), Campus Mukteswar, Nainital (Distt.), Uttarakhand, India; b Graphic Era University, Dehradun, Uttarakhand, India

## Abstract

We report the first complete genome sequence of a classical swine fever (CSF) virus of subgenotype 2.2. The virus (CSFV/IND/UK/LAL-290) was isolated from the Uttarakhand state of India from a backyard pig suspected of having CSF. This genome sequence will give useful insight for future molecular epidemiological studies and the development of an effective vaccine in India.

## GENOME ANNOUNCEMENT

Classical swine fever (CSF) is one of the notifiable viral diseases of swine worldwide, according to the World Organization for Animal Health (OIE). The disease causes severe economic losses worldwide, including in India, especially in the northeastern region where the pig population is higher. CSF is a highly contagious and lethal disease of pigs and wild boars caused by classical swine fever virus (CSFV). CSFV is a small enveloped virus encompassing a single-stranded RNA genome of positive polarity with a length of about 12.3 kb (1). The virus belongs to the genus *Pestivirus* in the family *Flaviviridae* and is structurally and antigenically related to other members of the genus *Pestivirus*, such as bovine viral diarrhea virus (BVDV) and border disease virus (BDV) (2). CSFV has one serotype divided into three major genotypes (1, 2, and 3) and 10 subgenotypes (1.1, 1.2, 1.3; 2.1, 2.2, 2.3; 3.1, 3.2, 3.3, and 3.4) (3). Based on phylogenetic analysis, the Indian isolates are grouped into two subgenotypes, 1.1 and 2.2, with a predominance of subgenotype 1.1 (4–6). In India, a solitary report of a CSFV whole-genome sequence is available, which belongs to the subgenotype 1.1 viruses (7).

In this study, 11 sets of oligonucleotide primers were designed, targeting overlapping fragments of CSFV from the 5′ to the 3′ end. Total RNA was isolated using Trizol reagent (Sigma-Aldrich, USA), and quality and concentration were checked with a Nanovue spectrophotometer. cDNA synthesis was carried out with a RevertAid first-strand cDNA synthesis kit (Thermo Scientific) employing a random hexamer primer. Both of the ends (3′ and 5′ untranslated regions [UTRs]) were amplified by gene-specific CSFV primers. The target regions were amplified into 11 overlapping fragments and cloned into a pJET1.2 blunt-end vector (Thermo Scientific). The recombinant plasmids were sequenced using pJET1.2 forward and reverse sequencing primers with an ABIPRISM 3100 genome sequencer. The sequences thus generated were annotated and analyzed using Lasergene 6 (DNASTAR) software. Further, to check the genotyping of the sample, phylogenetic analysis was performed with MEGA 5.1 software (8).

The sequences generated from 11 overlapping fragments of sample CSFV/IND/UK/LAL-290 covered the entire length of the 12,297 nucleotides (nt), including a 373-nt 5′ UTR, an 11,697-nt open reading frame (ORF) encoding a 3,898-amino-acid-long polyprotein, and a 227-nt 3′ UTR. Thus, this virus exhibited genomic organization analogous to that of other CSFV strains. The sequence alignment of CSFV/IND/UK/LAL-290 and the available CSFV whole-genome sequences (*n* = 54) in GenBank showed 82.0 to 91.1% identities at the nucleotide level and 87.9 to 92.5% identities at the amino acid level. A further sample of CSFV/IND/UK/LAL-290 showed 83.1 to 84.2%, 87 to 91.1%, and 82% similarities at the nucleotide levels and 88.3 to 90%, 91.2 to 92.5%, and 87.9% similarities at the amino acid levels to CSFV genotypes 1, 2, and 3, respectively. Sequence alignment revealed that this sample is genetically related to CSFV genotype 2 viruses, and phylogenetic analysis grouped this virus into subgenotype 2.2.

To the best of our knowledge, this is the first report of a whole-genome sequence of CSFV subgenotype 2.2 from the Uttarakhand state of India. The present study will help to make this virus a reference strain for the Indian subcontinent and will be useful for future molecular epidemiological investigations and the development of an effective vaccine in India.

### Nucleotide sequence accession number.

The whole-genome sequence of CSFV/IND/UK/LAL-290 has been submitted to GenBank under the accession number KC851953.
